# The Effect of COVID-19 on United States Pediatric Emergency Departments and Its Impact on Trainees

**DOI:** 10.5811/westjem.2022.7.57340

**Published:** 2022-10-18

**Authors:** Jessica Bailey, Nicole Nadeau, Kamyron Jordan, Hannah Yerxa, Samuel H.F. Lam

**Affiliations:** * Oregon Health & Science University, Department of Emergency Medicine, Portland, Oregon; † Massachusetts General Hospital, Department of Emergency Medicine, Boston, Massachusetts; ‡ Oregon Health & Science University, Department of Pediatrics, Portland, Oregon; § Sutter Medical Center Sacramento, Department of Emergency Medicine, Sacramento, California

## Abstract

**Introduction:**

The purpose of this study was to quantify the effects of the coronavirus disease 2019 (COVID-19) pandemic on pediatric emergency departments (PED) across the United States (US), specifically its impact on trainee clinical education as well as patient volume, admission rates, and staffing models.

**Methods:**

We conducted a cross-sectional study of US PEDs, targeting PED clinical leaders via a web-based questionnaire. The survey was sent via three national pediatric emergency medicine distribution lists, with several follow-up reminders.

**Results:**

There were 46 questionnaires included, completed by PED directors from 25 states. Forty-two sites provided PED volume and admission data for the early pandemic (March–July 2020) and a pre-pandemic comparison period (March–July 2019). Mean PED volume decreased >32% for each studied month, with a maximum mean reduction of 63.6% (April 2020). Mean percentage of pediatric admissions over baseline also peaked in April 2020 at 38.5% and remained 16.4% above baseline by July 2020. During the study period, 33 (71.1%) sites had decreased clinician staffing at some point. Only three sites (6.7%) reported decreased faculty protected time. All PEDs reported staffing changes, including decreased mid-level use, increased on-call staff, movement of staff between the PED and other units, and added tele-visit shifts. Twenty-six sites (56.5%) raised their patient age cutoff; median was 25 years (interquartile ratio 25–28). Of 44 sites hosting medical trainees, 37 (84.1%) reported a decrease in number of trainees or elimination altogether. Thirty (68.2%) sites had restrictions on patient care provision by trainees: 28 (63.6%) affected medical students, 12 (27.3%) affected residents, and two (4.5%) impacted fellows. Fifteen sites (34.1%) had restrictions on procedures performed by medical students (29.5%), residents (20.5%), or fellows (4.5%).

**Conclusion:**

This study highlights the marked impact of the COVID-19 pandemic on US PEDs, noting decreased patient volumes, increased admission rates, and alterations in staffing models. During the early pandemic, educational restrictions for trainees in the PED setting disproportionately affected medical students over residents, with fellows’ experience largely preserved. Our findings quantify the magnitude of these impacts on trainee pediatric clinical exposure during this period.

## INTRODUCTION

The severe acute respiratory syndrome coronavirus-2, cause of coronavirus disease 2019 (COVID-19), was first identified by the World Health Organization (WHO) in December 2019, and by March 2020 a worldwide pandemic was declared.^1^ With concerns for a strained medical system, many healthcare facilities instructed patients to seek medical attention for life-threatening emergencies only.^2^ Emergency departments (ED) nationwide began noting drastic drops in patient census.^3^,^4^,^5^ During the COVID-19 pandemic, academic hospitals faced the additional burden of maintaining quality medical trainee education amid numerous challenges, including potential disease exposure, declining patient volumes, and strict personal protective equipment (PPE) allocation.

Published studies have explored the consequences of COVID-19 on general ED operations and staffing as well as educational impacts within specific medical and surgical specialties.^6^,^7^,^8^ However, there has been little research regarding the experience of faculty and trainees working specifically within pediatric emergency departments (PED). Our objective in this study was to identify the impact of the early COVID-19 pandemic on PEDs in the United States (US). Specifically, we sought to describe the effects on physician staffing, trainee presence, and imposed restrictions on patient care, while additionally quantifying changes in patient volumes, admission rates, and patient age limits.

## METHODS

### Study Design

We conducted a cross-sectional survey of US PEDs. Subjects were eligible to participate if they served in a pediatric emergency administrative directorship role. We contacted pediatric emergency medicine (PEM) leaders via email solicitation through pediatric subgroups of the American College of Emergency Physicians and the Society for Academic Emergency Medicine. Study recruitment information was also posted on the PEM-EM listserv, a publicly accessed research and collaboration hub within the PEM online community. The survey links were provided to prospective participants a total of three times between October 2020–January 2021.

### Survey Development and Content

The 21-question survey was developed by a multicenter team of emergency physicians and pediatric emergency physicians with the aid of an academic research navigator. Study data was collected and managed using REDCap electronic data capture tools hosted at Oregon Health & Science University. Survey content asked for only aggregate data; thus, the institutional review board granted a waiver of informed consent. Participants provided monthly PED census and admission data for the study period (April–July 2020, deemed the “early pandemic” period) as well as a comparison period from the previous year (April–July 2019, deemed the “pre-pandemic” period). We focused additional survey questions on two areas of interest: alterations to departmental staffing and trainee clinical involvement.

### Statistical Analysis

Aggregate data was reviewed within the RedCap database. Survey answers were expressed as frequencies and proportions for categorical variables or means (+/− SD) or medians (+/− interquartile range [IQR]) for continuous variables. Changes in pre-pandemic and early pandemic values were calculated and expressed as percentages. We performed statistical analyses using Microsoft Excel (Microsoft Corporation, Redmond, WA).

## RESULTS

There were 47 completed questionnaires from PED directors in 25 US states (Table). One hospital duplication was noted on manual ZIP code review; in this case, only the survey completed by the more senior leadership was included for 46 questionnaires total. We were unable to calculate target audience response rate given the mixed nature of distribution list recipients, with PED directors representing only a small and unspecified number of their subscribers. The median annual volume was 20,001–30,000. Surveyed sites were a mix of freestanding children’s hospitals, general academic/university medical centers, and community hospitals.

Pre-pandemic, surveyed PEDs reported having treated patients up to 17–22 years of age. During the early COVID-19 pandemic, 26 sites (56.5%) raised their patient age cutoff, to a median of 25 years (IQR 25–28 years). All PEDs reported staffing changes of some kind. Only three sites (6.7%) reported decreased faculty protected time. (See Appendix A for detailed PED operational responses.)

Forty-two sites provided PED volume and admission data for the months of March–July 2020 and March–July 2019. Mean percentage changes over time are presented in the Figure. The largest mean PED volume change occurred in April 2020, with a decrease of 63.6% compared to April 2019. At the same time, increase in mean admission rate peaked in April 2020 with a rise of 38.4% compared to April 2019. These trends continued, although they were less pronounced through the end of the study period in July 2020 with mean volumes down 33.5% and mean admission rates up 16.4% from the prior year.

Forty-four (95.7%) of the sites reported hosting trainees (medical students, residents, fellows) during normal operating times. (See Appendix B for detailed PED education responses.). Of those, 36 (78.3%) reported a decrease in numbers of trainee or eliminating trainees altogether (70.5% and 11.4%, respectively), and 21 sites (47.7%) reported decreased total ED hours for the trainees who they did maintain. Thirty (68.2%) of the sites had restrictions on patient care provision by trainees. Of these, 28 sites (63.6%) placed restrictions on medical students, 12 sites (27.3%) restricted residents, and only two sites (4.5%) restricted fellows. A minority of the sites (15, 34.1%) placed specific restrictions on procedures performed by trainees: medical students (13, 29.5%); residents (9, 20.5%); and fellows (2, 4.5%) (Appendix B).

## DISCUSSION

In this retrospective survey of US PEDs, we found that patient volume dropped precipitously while percentages of patients admitted increased considerably during the early stages of the COVID-19 pandemic. A significant proportion of surveyed PEDs also implemented restrictions on patient care by medical trainees, limiting their educational experiences. The timing and magnitude of PED volume drop in our study is consistent with previously published studies from the US and around the world during the pandemic.^9^,^10^,^11^ In many cases, the census decline occurred despite increased PED patient age cut-offs. On the other hand, the percentage of PED admissions increased during the same period. As a result of decreased patient volumes, the staffing model of many PEDs changed. Fortunately, protected time for PED faculty remained relatively intact, with only four sites reporting a temporary reduction in research, education, or administrative hours.

Our study provides valuable insight into how decreasing patient volumes and safety concerns associated with the pandemic impacted the clinical experience for medical students, residents, and fellows rotating through US PEDs. Of significance, we found that while trainees of all levels were appreciably influenced by the COVID-19 pandemic, the repercussions for medical students were the most significant. On March 17, 2020, the Association of American Medical Colleges issued guidelines strongly supporting medical schools pausing clinical rotations for medical students.^12^ In addition to their decreased numbers or complete elimination from many PED sites, most remaining students were restricted in their provision of patient care or procedures performed. A recent survey found that most US medical students felt that removal from clinical rotations was appropriate but that it resulted in decreased opportunity to develop skills needed for residency.^13^ It remains to be seen whether PED patient restrictions during this period will impair future residency choice or interest in PEM as a subspecialty.

While there have been published studies exploring the effects of the COVID-19 pandemic on surgery, radiology, and neurosurgery residents, there is much less data on its impact on US EM residents.^14^,^15^,^16^ For many EM residents, PED rotations represent the bulk of their pediatric clinical exposure during residency. Access to the full spectrum of pediatric cases by EM residents is widely variable across programs, and a large portion of residents demonstrate deficits in their exposure to common pediatric diagnoses by completion of training.^17^ In our study, 27.3% of the PEDs reported restriction in residents providing patient care and 20.5% reported restriction in residents performing procedures.

It would follow that the pandemic-related decrease in PED patient volume and restrictions on care provision during those rotations may have further limited EM residents’ opportunities for pediatric-specific training. It is currently unknown whether these challenges could additionally affect physician levels of confidence in treating future pediatric patients presenting to the ED. Individual programs would benefit from careful review of the pandemic period’s impact on their resident caseloads and consider expanding opportunities for other learning modalities (such as didactics, case-based discussions, or simulations) to address any potential clinical knowledge gaps in pediatrics.

## LIMITATIONS

There are several limitations to this study. With a voluntary survey method, the possibility of selection bias exists, in that physicians may have been more likely to complete the survey if they felt their departments had been significantly altered by the pandemic. In addition, the data was physician-reported and hence unverified. Finally, while we made significant efforts to obtain a nationally representative sample and obtained data from 25 states and all categories of practice settings, the survey response was somewhat modest and may not be entirely representative of the experience of all US PEDs.

## CONCLUSION

This survey provided a cross-sectional perspective of the impact the early portion of the COVID-19 pandemic had on US pediatric emergency departments. Medical trainee education was affected by a sustained drop in pediatric ED volumes as well as institutional restrictions on patient care and procedure involvement, with medical students being disproportionately affected. Our study highlights the dynamic challenges PEDs face during a public health emergency and suggests that additional attention is needed to ensure medical learners receive the support and clinical experience they require during such unprecedented times.

## Supplementary Information



## Figures and Tables

**Figure 1:**
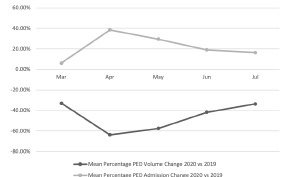
Mean pediatric emergency department volume and admission percentage changes over time. *PED*, pediatric emergency department.

**Table:** Characteristics of survey respondents.

Variable	Number (percentages)
State (N = 46)	
CA, FL, IL	5 (10.9) each
MN, NY	3 (6.5) each
NC, OR, PA, TN, TX	2 (4.3) each
AZ, CT, IA, ME, MA, MD, MI, NV, NJ, NM, OH, OK, RI, WA, WI	1 (2.2) each
PED setting (N = 46)	
Academic/ university center	17 (37.0)
Freestanding children’s hospital	12 (26.1)
Community-based hospital	11 (23.9)
Combined adult and pediatric emergency departments	5 (10.9)
Other	1 (2.2)
PED annual volume (N = 46)	
< 10,000 patients per year	2 (4.3)
10,001–20,000 patients per year	13 (28.2)
20,001–30,000 patients per year	11 (23.9)
30,001–40,000 patients per year	3 (6.5)
>40,000 patients per year	17 (37.0)

*PED*, pediatric emergency department.
