# Supplementary material for: The Effect of COVID-19 on United States Pediatric Emergency Departments and Its Impact on Trainees
Source: West J Emerg Med. 2022 Oct 18;23(6):893–6. doi: 10.5811/westjem.2022.7.57340 (PMC9683764; doi:10.5811/westjem.2022.7.57340)
Supplement: Supplementary file 1 [file wjem-23-893-s001.docx]

APPENDIX A:

Detailed breakdown of pediatric emergency department operations-related survey responses.

| **Survey Question** | | **Number (percentage)** |
| --- | --- | --- |
| **In normal operating times, what is your PED maximum age cutoff? (N = 46)** | | |
| Mean |  | 19.1 years (SD 1.54 years) |
| Median |  | 18 years (range 17-21 years) |
| **At any point during the COVID-19 pandemic (after March 1, 2020) did you change your maximum age cut-off? (N = 46)** | | |
| Yes |  | 26 (56.5) |
| Mean |  | 29 years (SD 15.0 years) |
| Median |  | 25 years (range 21-95 years) |
| No |  | 20 (43.4) |
| **Since March 1, 2020, in response to COVID-19: Has your PED increased/ decreased physician staffing (ie, assigned clinical shift) hours at any point? (n = 45)** | | |
| Increased |  | 1 (2.2) |
| Decreased |  | 32 (71.1) |
| No change |  | 12 (26.7) |
| **Since March 1, 2020, in response to COVID-19: What other change in staffing has been implemented in your PED? (n = 45)** | | |
| Added tele/video visit shifts |  | 2 (4.4) |
| Decreased use of mid-levels |  | 20 (44.4) |
| Increased use of mid-levels |  | 0 (0) |
| Additional on-call phycians/staff |  | 14 (31.1) |
| Transition of PED physicians to/ from other hospital units | | 9 (20.0) |
| No other change |  | 12 (26.7) |
| **Since March 1, 2020, in response to COVID-19: Has your department/ division made any adjustments in provider/faculty protected time (eg, contracted time set aside for education, research, or administrative duties)? (n =45)** | | |
| Yes |  | 3 (6.7) |
| Education |  | 0 (0) |
| Research |  | 1 (2.2) |
| Administration |  | 3 (6.7) |
| No |  | 42 (93.3) |
| **For how long are/were these changes to protected time in place? (n = 3)** | | |
| < 12 weeks |  | 1 (33.3) |
| 3-6 months |  | 1 (33.3) |
| 7-12 months |  | 0 (0) |
| Undetermined at this time |  | 1 (33.3) |

APPENDIX B:

Detailed breakdown of pediatric emergency department education-related survey responses.

| **Survey Question** | | **Number (percentage)** |
| --- | --- | --- |
| **During normal operating times, do you have trainees rotating through your PED? (N = 46)** | | |
| Yes |  | 44 (95.7) |
| No |  | 2 (4.3) |
| **During normal operating times, which trainees rotate through your PED? (n = 44)** | | |
| Medical students |  | 37 (84.1) |
| Residents from own institution |  | 38 (86.3) |
| Residents from outside institution |  | 32 (72.7) |
| Fellows from own institution |  | 23 (52.3) |
| Fellows from outside institution |  | 3 (6.8) |
| **At any time during March 2020 - July 2020, did your PED experience a change in the numbers of rotators, different from expected numbers during normal operation? (n = 44)** | | |
| Increase in number |  | 0 (0) |
| No change in number |  | 8 (18.2) |
| Reduction in number |  | 31 (70.5) |
| Elimination altogether |  | 5 (11.4) |
| **At any time during March 2020 - July 2020, did your PED experience a change in hours for any of the rotators? (n = 44)** | | |
| Reduction in hours |  | 21 (47.7) |
| Increase in hours |  | 0 (0) |
| No change in hours |  | 23 (52.3) |
| Eliminated |  | 1 (2.3) |
| **Since March 1, 2020, in response to COVID-19: Has your PED or institution placed any restrictions on the provision of patient care by trainees (eg, types of patients they can care for)? (n = 44)** | | |
| Yes |  | 30 (68.2) |
| Restrictions for medical students |  | 28 (63.6) |
| Restrictions for residents |  | 12 (27.3) |
| Restrictions for fellows |  | 2 (4.5) |
| No |  | 14 (31.8) |
| **Since March 1, 2020, in response to COVID-19: Has your PED or institution placed restrictions on procedures trainees are allowed to perform? (n = 44)** | | |
| Yes |  | 15 (34.1) |
| Restrictions for medical students |  | 13 (29.5) |
| Restrictions for residents |  | 9 (20.5) |
| Restrictions for fellows |  | 2 (4.5) |
| No |  | 29 (65.9) |

*PED*, pediatric emergency department.
